# First description of the male of *Hiranetis atra* Stål and new country records, with taxonomic notes on other species of *Hiranetis* Spinola (Hemiptera, Heteroptera, Reduviidae, Harpactorinae)

**DOI:** 10.3897/zookeys.605.8797

**Published:** 2016-07-14

**Authors:** Hélcio R. Gil-Santana

**Affiliations:** 1 Laboratório de Diptera, Instituto Oswaldo Cruz, Av. Brasil, 4365, 21040-360, Rio de Janeiro, RJ, Brazil

**Keywords:** Costa Rica, Ecuador, Graptocleptes, Harpactorini, Hiranetis braconiformis, Hiranetis membranacea, wasp-mimicking bug

## Abstract

The male of *Hiranetis atra* Stål, 1872 is described and illustrated for the first time. In addition, this paper illustrates the female and provides new country records for this species. Photographs of all extant types of species of *Hiranetis* Spinola, 1840 are presented with taxonomic notes on the other two species of the genus.

## Introduction

Harpactorinae is the largest subfamily of Reduviidae and is represented by the tribes Apiomerini and Harpactorini in the Neotropical region (Gil-Santana et al. 2015). Harpactorini is the most diversified Reduviidae group with more than 53 recognized genera in the Neotropical region (McPherson and Ahmad 2011, Forero 2011, 2012, Swanson 2012, Gil-Santana 2015, Gil-Santana et al. 2015). The only outdated key to American Harpactorini genera is that of Stål (1872). However, Maldonado and Lozada (1992) presented a key to Neotropical wasp-mimetic Harpactorinae genera, which in their view helps to quickly sort out specimens from unidentified material, although this is a somewhat artificial way of grouping genera. Maldonado and Lozada (1992) considered six Neotropical Harpactorini genera to be wasp-mimetic: *Acanthischium* Amyot & Serville, 1843, *Graptocleptes* Stål, 1866, *Hiranetis* Spinola, 1840, *Myocoris* Burmeister, 1835, *Neotropiconyttus* Kirkaldy, 1909 and *Xystonyttus* Kirkaldy, 1909. They regarded *Neotropiconyttus* as resembling braconids, while all others somewhat resembled ichneumonid wasps. Although *Coilopus* Elkins, 1969 was described as a wasp-mimicking genus (Elkins 1969), Maldonado and Lozada (1992) considered it akin to bees and did not include this genus in their key. Gil-Santana (2015) has updated this key, including all these seven genera, and also *Parahiranetis* Gil-Santana, 2015. Forero and Giraldo-Echeverry (2015) further proposed that a Vespidae (*Mischocyttarus* sp.) was the hymenopteran mimetic model of *Coilopus vellus* Elkins, 1969.

Recently, Gil-Santana et al. (2013) showed that *Hiranetis coleopteroides* (Walker, 1873) was in fact a species of *Graptocleptes* and a junior synonym of *Graptocleptes bicolor* (Burmeister, 1838). Therefore, three species are currently included in *Hiranetis*: *Hiranetis atra* Stål, 1872, *Hiranetis braconiformis* (Burmeister, 1835) and *Hiranetis membranacea* Spinola, 1840 (Maldonado 1990, Gil-Santana et al. 2013).

Champion (1898) considered *Hiranetis* spp. to resemble various Ichneumonidae and Braconidae (Hymenoptera), while Haviland (1931) recorded a Müllerian mimicry association among species of *Graptocleptes*, and an association between *Xystonyttus* and ichneumonid wasps. Hogue (1993) cited a similar association among species of *Graptocleptes* and *Hiranetis*.

In a review of *Alabagrus* Enderlein, 1920 (Hymenoptera: Braconidae), Leathers and Sharkey (2003) argued that many species of this genus belong to a Neotropical, presumably mimetic complex, with thousands of other species, including 1,300 species of Braconidae in other genera, more than 1,000 species of Ichneumonidae, several hundred species of Reduviidae (e.g. *Hiranetis*) and unknown numbers of species in other orders. Some of the Reduviidae, the ‘braconiformes clade’, have wings, shape and physical proportions that are very similar to some braconids (Leathers and Sharkey 2003). These authors presented a photo of a specimen in lateral view, identified as Hiranetis nr. braconiformis (Burmeister, 1835), to illustrate their assertion.

Hespenheide (2010) recorded examples of mimicry of braconids by *Agrilus* Curtis, 1825 (Coleoptera: Buprestidae). In Panama, species of *Agrilus* share a braconid-like color pattern with the orders Coleoptera, Diptera and Hymenoptera, and with six species of Reduviidae, including Hiranetis nr. braconiformis and five other undetermined species.

Most authors have only mentioned or taken into consideration the pattern of yellowish or straw-colored hemelytra with a median transverse black band, in relation to the alleged mimicry between Harpactorini and certain Ichneumonidae and Braconidae, as models (Champion 1898, Haviland 1931, Maldonado and Lozada 1992, Hogue 1993, Leathers and Sharkey 2003, Hespenheide 2010). On the other hand, Gil-Santana (2015) has emphasized that other wasp-mimicking Harpactorini, like *Parahiranetis salgadoi* Gil-Santana, 2015, show a pattern of darkened to reddish general colouration with yellowish ‘pterostigmata’ on the hemelytra, which is similar to the coloration also exhibited by several other species of Ichneumonidae and Braconidae. This pattern was also observed for instance in *Graptocleptes bicolor* and *Graptocleptes haematogaster* (Stål, 1860). Another common feature among all these Harpactorini species with a darkened general coloration on the hemelytra, including in *Hiranetis atra* and *Graptocleptes sanguiniventris* (Stål, 1862), is a yellowish band on the femora (Gil-Santana 2015).

Sexual dimorphism has been recorded in several species of Harpactorini. In addition to the bigger size and larger abdomen of females, which is common in many other insects, males in several genera have larger eyes and/or the thickening of the third antennal segment in its basal portion. The latter has been considered to be among the diagnostic features at genus level (Stål 1872, Champion 1898, Gil-Santana et al. 2013, Martin-Park et al. 2012).

Champion (1898) recorded that the males of *Hiranetis braconiformis* present thickening of the third antennal segment at its base and, apparently based only on this species, stated that this was a feature belonging to *Hiranetis*.

In the present paper, the male of *Hiranetis atra* is described and illustrated for the first time. In addition, this paper illustrates the female and provides new country records for this species. Photographs of all extant types of species of *Hiranetis* and taxonomic notes on the other two species of the genus are presented.

## Material and methods

Photographs of the type specimens of *Hiranetis atra*, which are deposited at the Swedish Royal Natural History Museum
(NRM), Stockholm, Sweden, were made by Dr Gunvi Lindberg (NRM). The other extant types and additional specimens were directly examined. The respective depositories and curators, who kindly allowed me to examine them, are the following: “Museum für Naturkunde der Humboldt-Universität zu Berlin” (ZMHB), Berlin, Germany, Dr Jürgen Deckert, and “Muséum National d’Histoire Naturelle” (MNHN), Paris, France, Dr Éric Guilbert.

Dissections of the male genitalia were made removing the pygophore from the abdomen with a pair of forceps and then clearing it in KOH solution for 24 hours. The dissected structures were studied and photographed in glycerol. Drawings were made using a *camera lucida*. Images of external and genital structures by the author were taken with digital cameras (Nikon D5200® with a Nikon® Macro Lens 105 mm, Sony DSC-W830® and Sony DSC-HX400V®). The vestiture (setation) was omitted in the ink drawings showing some genital structures (Figs 7–8) in order to make more clear the shape and/or structure of these areas. General morphological terminology mainly follows Schuh and Slater (1995). Terminology applied to male genital characteristics follows mainly those used by Gil-Santana et al. (2013). Measurements are in millimeters (mm).

## Taxonomy

### Hiranetis

Taxon classificationAnimaliaHemipteraReduviidae

Spinola, 1840

Hiranetis Spinola, 1840: 112–113 [description]; Stål 1859: 367 [key], 371 [citation, species included]; Stål 1866: 294 [key]; Stål 1872: 69 [diagnosis, key], 82–83 [catalog]; Walker 1873a: 64 [key] ; Walker 1873b: 129 [catalog]; Lethierry and Severin 1896: 178 [catalog]; Champion 1898: 280 [comments]; Wygodzinsky 1949: 40 [catalog]; Elkins 1969: 459 [citation]; Putshkov and Putshkov 1985: 46 [catalog]; Maldonado 1990: 218 [catalog]; Maldonado and Lozada 1992: 165 [key]; Froeschner 1999: 206 [catalog]; Forero 2011: 15 [checklist]; Gil-Santana et al. 2013: 348, 358 [citations], 359 [separation from Graptocleptes]; Gil-Santana 2015: 29, 30 [citations], 35, 36 [separation from Graptocleptes and Parahiranetis], 37 [key].

#### Type species.

*Hiranetis membranacea* Spinola, 1840: 113–114, by monotypy.

#### Diagnosis.

General appearance: wasp-mimetic. Head gibbous, large, as long as wide across eyes, densely covered with long setae on ventral and postocular portions; postantennal tubercles very short to almost imperceptible, acute or rounded; legs elongated, slender; fore femur slightly longer than head and pronotum together, thicker basally. Hemelytra long, surpassing the abdomen by about half of the length of the membrane.

### Hiranetis atra

Taxon classificationAnimaliaHemipteraReduviidae

Stål, 1872

Figures 1–5 , 6–10 , 11–13 , 14–17

Hiranetis atra Stål, 1872: 82–83 [description]; Lethierry and Severin 1896: 178 [catalog]; Wygodzinsky 1949: 40 [catalog]; Maldonado 1990: 218 [catalog]; Gil-Santana et al. 2013: 348 [citation]; Gil-Santana 2015: 36 [citation].

#### Notes.

*Hiranetis atra* was first described based on one or more female specimens from Bogotá, Colombia (Stål 1872), without any further descriptions of the species. It is noteworthy that although the type locality of *Hiranetis atra* might really be “Bogota”, it is possible that the real locality of collecting of the specimens had been different. In the 19^th^ century, “Bogotá” was just the shipping denomination for the commercial trade, including specimens going to Europe (Forero 2006).

Although no figures of *Hiranetis atra* have so far been published, the Swedish Royal Natural History Museum (NRM) has made photos of its type available, and these can be freely accessed at: http://www2.nrm.se/en/het_nrm/a/hiranetis_atra.html.

Based on these photos, Gil-Santana (2015) stated that *Hiranetis atra* would have very small yellowish markings like dots in hemelytra, at a site where some other wasp-mimicking Harpactorini have larger yellowish ‘pterostigmata’.

**Figures 1–5.**
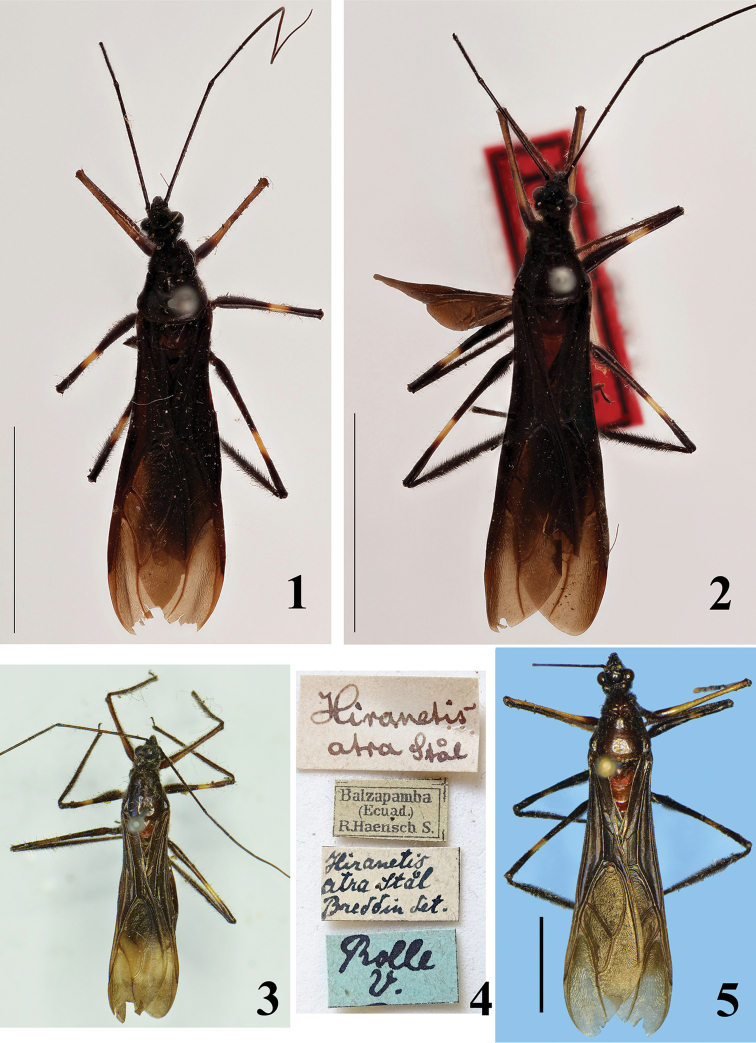
*Hiranetis atra* Stål, females. **1–2** syntypes, dorsal view, photos: Gunvi Lindberg. Copyright Swedish Museum of Natural History, Stockholm (NRM). Scale bars: 10 mm. **1** “type”, **2** “paratype” **3–4** specimen from Ecuador deposited in ZMHB **3** dorsal view **4** labels **5** specimen from Costa Rica, deposited in MNHN, dorsal view. Scale bar: 5.0 mm.

However, Dr Gunvi Lindberg (NRM) subsequently provided new figures (Figs 1–2) and the information that both “type” and the “paratype” of *Hiranetis atra* have hemelytra completely dark. It seems that the apparent small dot on the hemelytra is likely to be some form of fouling, like mycelium.

On the other hand, because the original description (Stål 1872) did not mention the number of types or designate a holotype, as was generally done at that time, it is better to consider all the type specimens to be syntypes.

In addition to the male and female from Costa Rica that are described below, an additional female from Ecuador (Figs 3–4) was examined at ZMHB, where it is deposited.

#### Material examined.

One male and one female, each with a green label with the same information: “Museum Paris, Costa Rica, Paul Serre, 1920” (MNHN). One female, labels: *Hiranetis atra* Stål / Balzapamba, (Ecuad.), R.Haensch S. / *Hiranetis atra* Stål, Breddin det. / k[?]olle v. [green label] (ZMHB).

#### Diagnosis.

**Figures 6–10.**
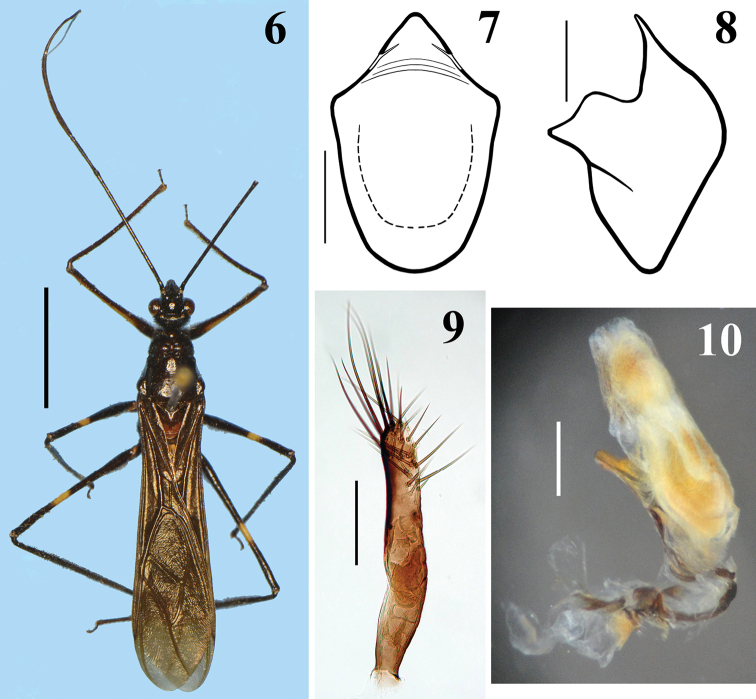
*Hiranetis atra* Stål, male from Costa Rica, deposited in MNHN. **6** dorsal view **7–8** pygophore without parameres. **7** ventral view **8** lateral view **9** paramere. **10** phallus, lateral view. Scale bar: (**6**): 5.0 mm; (**7–8, 10**): 0.5 mm; (**9**): 0.2 mm.

*Hiranetis atra* can be readily separated from other species of the genus by its general coloration, which is mostly blackish, especially the hemelytra, which are completely dark (Figs 1–3, 5–6), while the other species have the pattern of yellowish or straw-colored hemelytra, with a median transverse band and dark apex.

#### Description.

**Figures 11–13.**
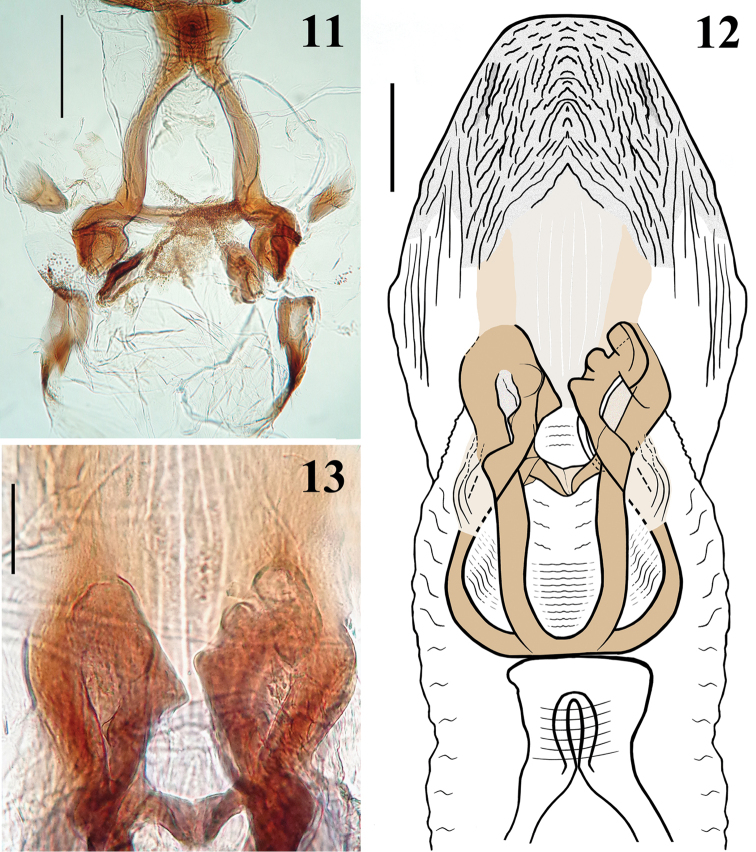
*Hiranetis atra* Stål, male genitalia, dorsal view. **11** articulatory apparatus **12** basal plate extension (pedicel), phallothecal dorsal plate and struts **13** struts apices. Scale bar: (**11**): 0.3 mm; (**12**): 0.2 mm; (**13**): 0.1 mm.

**Figures 14–17.**
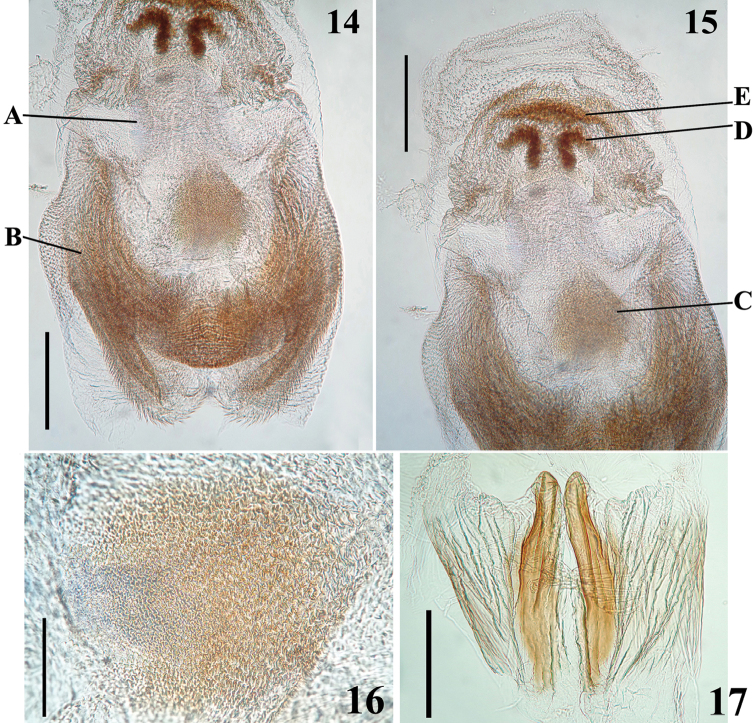
*Hiranetis atra* Stål, male genitalia, dorsal view. **14–15** endosoma, without dorsal apical-median process. (A: semi-oblong dorsal prolongation at midportion of endosoma wall; B–E: endosoma processes, B: U to M-shaped basal process; C: median subspherical process; D: small sclerotized thickened curved process; E: transverse thickening) **16–17** endosoma processes **16** median. **17** dorsal apical-median. Scale bar: (**14–15**): 0.3 mm; (**16**): 0.1 mm; (**17**): 0.3 mm.

MALE. Figures 6–17. Measurements (mm): Total length: to tip of abdomen: 12.1; to tip of hemelytra: 16.2; head: total length (lateral view): 1.9; maximum width across eyes: 1.9; interocular space: 1.0; antennal segments: I: 5.5; II: 1.7; III (very bent; approximately): 6.9; IV: 2.0; labium segments: II [first visible]: 1.4; III: 1.1; IV: 0.3. Thorax: pronotum: fore lobe length: 0.7; hind lobe: length: 2.0; width at posterior margin: 2.8. Legs: fore legs: femur: 5.4; tibia: 5.5; tarsus: 0.7; mid legs: femur: 4.8; tibia: 6.2; tarsus: 0.7; hind legs: femur: 6.5; tibia: 9.1; tarsus: 0.8. Abdomen: length: 6.3; maximum width: 2.3. Coloration: general coloration black (Fig. 6). Head, including antennae and labium, blackish, eyes brownish-black. Thorax blackish, with exception of metanotum, which is reddish-brown. Hemelytra blackish. Legs mostly blackish; fore femur with dorsal surface, except at base and extreme apex, pale yellowish, and with a lighter-colored subbasal portion ventrally; mid and hind femora with yellowish annulus situated somewhat distally to their midportion (Fig. 6). Abdominal segments II and III (first two visible) reddish; sternite IV almost completely reddish, except on posterolateral portion, including connexivum at this area, where it is blackish; sternite V mostly reddish but blackish on posterior and lateral portion, including connexivum. Tergites IV and V, and remaining segments, including pygophore and parameres, blackish. Structure and vestiture: Integument mostly shiny, smooth. Head gibbous, large, as long as wide across eyes; integument shiny, with sparse long and short, straight or somewhat curved blackish setae; the latter much denser, forming pubescence of long blackish thick setae on postocular portion and gula; almost completely glabrous between eyes. Labium curved, with scattered and somewhat curved blackish setae. Antennal segments I and II straight, the former approximately three times longer than head, with shiny and smooth integument and sparse short darkened setae; segments II-IV with opaque and somewhat rugose integument; segment II, except at basis, covered with very numerous darkened short setae, with some longer intermixed setae and some very thinner elements at distal portions (interpreted as trichobothria); segment III thickened in basal half, curved; III and IV covered with dense, very short and somewhat lighter-colored pubescence, with short darkened setae scattered on segment III and few of these on segment IV; the latter is thinner than the other segments and moderately curved. Postantennal tubercles small and somewhat acuminate. Eyes globose, glabrous, projecting laterally, prominent in dorsal view, reaching dorsal margin of head at interocular sulcus in approximately its midportion; not reaching ventral margin of head, which is far from inferior margin of the eye. Interocular sulcus thin and moderately deep. Ocelli elevated, much closer to eyes than to each other. Collum thin. Thorax with shiny integument; prothorax covered with very numerous blackish thick setae on forelobe, anterior portions of propleura and hind lobe; the latter with sparse long setae at dorsal portion or, almost glabrous, except on midline, where thinner, somewhat shorter and light yellowish to whitish setae form a faint midlongitudinal line on hind lobe. Transverse sulcus not very deep, interrupted before middle by a pair of submedian shallow carina. Midlongitudinal sulcus on forelobe of pronotum becoming abruptly deeper at transverse sulcus to form a depression; posteriorly to the latter, a blunt short rounded prominence; disc of hind lobe smooth; lateral longitudinal sulci well marked at posterior half to posterior two-thirds of hind lobe of pronotum. Humeral angle elevated, rounded at lateral margin; median portion of posterior margin of pronotum with some long thin darkened setae. Scutellum elevated at disc, pointed posteriorly, with scattered thin dark long setae. Posterior portion of propleura, mesopleura, metapleura and thoracic sterna with long darkened setae, which are shorter and thinner at center of mesosternum and metasternum. Legs: coxae with numerous long dark setae on distal half, ventrally, and some longer thinner light-colored elements, while the basal third and lateral portions are almost completely glabrous; trochanters densely covered with long setae ventrally and with some scattered even longer thinner setae, which are lighter-colored on forelegs and dark on mid and hind legs. Fore femur subequally longer than head and pronotum together; all femora thicker basally and slightly subapically too, covered with scattered few long and strong dark setae and with a dense group of long and thick setae and some thinner and even longer setae on ventral portion of the basal enlarged portion of femora; these setae are lighter on fore femora and darker on the others. Fore tibiae somewhat curved, with uniform thickness, except at apex, which is somewhat enlarged, and where there is a dorsal spur and a mesal comb. Mid and hind tibiae straight and somewhat thickened at basal half. All tibiae with scattered long thick blackish setae; fore and mid tibiae covered with shorter dark setae on ventral surface, which become progressively more numerous towards apex, where they also covers lateral and dorsal surfaces; hind tibiae, except at base, densely covered with short dark setae, which are somewhat longer in the slightly enlarged basal half. Tarsi with moderately long dark setae. Hemelytra long, surpassing abdomen by about half length of membrane; corium with curved scattered adpressed short dark setae, which are much more numerous over costal and subcostal veins, becoming less numerous on distal half of corium, including over those veins; membrane glabrous. Abdomen: elongate; spiracles rounded; sternites with shiny integument and sparse long thin setae, which are light on reddish portions and dark on the blackish segments, and thicker, longer and more numerous on parts adjacent to genitalia and on the latter too. There is also a fusiform grouping of whitish minute short setae on midlateral portions of sternite V. MALE GENITALIA (Figs 7–17): pygophore: blackish, subpentagonal in ventral view, with a subtriangular rounded apex (medial process) (Fig. 7); lateral to the latter, a somewhat deep and rounded emargination (Fig. 8); between anterior and genital opening, a very well sclerotized bridge that has a conspicuous median dorsal rounded prominence; long, thick and dark setae ventrally (on exposed surface), somewhat more numerous on lateroapical portions. Parameres symmetrical, rod-like in shape; somewhat curved in basal half and straight towards apices, which are rounded, blackish, glabrous in basal two-thirds and with long setae in apical third; those at apicomedial margins even longer (Fig. 9). Phallus somewhat elongate when not inflated (Fig. 10); articulatory apparatus with basal plate arms and bridge narrow, forming a subrectangular set, except in apical portion, where the arms are curved (Fig. 11); basal plate extension (pedicel) moderately short, slightly expanded towards apex and somewhat more sclerotized than the arms and basal bridge (Fig. 11). Dorsal phallothecal plate weakly sclerotized, flat, suboval in shape, with numerous longitudinal thin grooves at apical half; apical margin almost transverse, straight (Fig. 12). Struts with curved lateral arms and parallel somewhat curved median arms which are expanded at apex into a pair of asymmetrical sub oval/subsquared structures; there is a medial bridge joining the bases of the latter (Figs 12–13). Endosoma wall mostly minutely spiny, with a small smooth semi-oblong dorsal prolongation at midportion (Figs 14–15). After endosoma extension, seven processes were observed: 1 - a larger U to M-shaped basal process formed by diffuse thickening (Fig. 14); 2 - a median subspherical process, situated between the upper arms of the basal process, formed by minute tooth-like thickenings (Figs 14–16); 3 - a pair of elongate apical-median flat, longitudinally striated and somewhat curved and moderately sclerotized processes, wrapped in a smooth portion (not minutely spiny, but with fine longitudinal grooves) of endosoma wall, all of which lying dorsally to the other subapical processes described next (Fig. 17); 4 - a pair of small sclerotized thickened curved processes, located below the next process (Fig. 15); 5 - a transverse thickening above the pair of processes described previously (Fig. 15). The spiny endosoma wall above the latter process has larger and more sclerotized elements (Fig. 15).

FEMALE (from Costa Rica): Measurements (mm): Total length: to tip of abdomen: 16.5; to tip of hemelytra: 21.0; head: total length (lateral view): 2.3; maximum width across eyes: 2.2; interocular space: 1.2; antennal segments: I: 6.3; II: 2.3; III-IV: absent; labium segments: II [first visible]: 1.6; III: 1.3; IV: 0.5. Thorax: pronotum: fore lobe length: 0.9; hind lobe: length: 2.5; width at posterior margin: 3.5. Legs: fore legs: femur: 6.0; tibia: 6.0; tarsus: 0.7; mid legs: femur: 4.9; tibia: 6.5; tarsus: 0.7; hind legs: femur: 7.2; tibia: 10.0; tarsus: 0.9. Abdomen: length: 9.5; maximum width: 3.8. Similar to male (Fig. 5). Anterior half of stridulitrum lighter-colored, reddish; sternite IV completely reddish; sternite V almost completely reddish, except on posterolateral portion, including connexivum in this area, where it is blackish; mid-anterior portion of sternite VI somewhat reddish.

#### Comments.

Since all the specimens studied here have hemelytra that are completely darkened without any yellowish markings (Figs 1–3, 5–6), the mistake in the statement of Gil-Santana (2015), who alleged the presence of small yellowish markings on the hemelytra, is confirmed. Because the features of females examined are in accordance with the description (Stål 1872) and with those of the syntypes of *Hiranetis atra* (Figs 1–2), they were considered conspecific. Similarly, the male collected together with the female from Costa Rica was considered as belonging to the same species too. The variation in size, in which the male was shown to be smaller than the female measured here, may or may not be due to sexual dimorphism. This would be clarified if or when more specimens of both sexes are examined in the future. Additional data might also show whether the eyes of the males are or are not larger in this species, since it was not possible to ascertain this through the single observation made here. Although the third antennal segments were absent in the female that was directly compared with the male that had been collected together with it (from Costa Rica; Figs 5–6), the other females recorded here (Figs 1–3) show uniform thickness in this segment, while the male presented thickening in the basal half of this segment (Fig. 6). This form of sexual dimorphism has been recorded in several genera of Harpactorini (Stål 1872, Champion 1898, Gil-Santana et al. 2013, Martin-Park et al. 2012) and in another species of *Hiranetis*, *Hiranetis braconiformis* (Champion 1898). The minor differences in coloration between the male and female examined were probably due to intraspecific variation, as already recorded in other species of *Hiranetis* (Spinola 1840, Herrich-Schäffer 1848, Champion 1898). On the other hand, they are in accordance with the Stål’s concise description of *Hiranetis atra*, including the coloration of the abdomen, which he defined as reddish in its basal half. The total length (measured to the tip of the abdomen) of the female described by Stål was 22 mm, i.e. very similar to that of the female specimen examined here (21 mm).

The importance of the male genitalia for distinguishing species within Harpactorini genera has previously been recorded, e.g. in *Aristathlus* Bergroth, 1913 (Forero et al. 2008), *Atopozelus* Elkins, 1954 (Elkins 1954a), *Atrachelus* Amyot & Serville, 1843 (Elkins 1954b), *Ischnoclopius* Stål, 1868 (Hart 1975) and *Zelus* Fabricius, 1803 (Hart 1972, 1986, 1987, Zhang 2012). For the latter, which is a very speciose genus, studying the male genitalia for taxonomic purposes was shown to be so important that “while males of most species [of *Zelus*] can be readily identified based on characters of the genitalia, identification of females is less straightforward” (Zhang 2012). In all of these studies, the main structures that were shown to be important or that had attributes at a specific level were the medial process of the pygophore, the dorsal phallothecal plates and the struts. The endosoma contents, such as its processes, were not examined or recorded in most of these studies. Although other authors have provided records regarding endosomal structures, most of these studies relate to a single species or very few species in different genera of Neotropical Harpactorini, e.g. *Aristathlus* spp. (Forero et al. 2008), *Graptocleptes bicolor* (Gil-Santana et al. 2013) and *Pronozelus schuhi* Forero, 2012 (Forero 2012). This impedes comparative appraisal between the studies for taxonomic purposes.

There are no previous studies describing the male genitalia of any species of *Hiranetis*, but there is one study on a species of *Graptocleptes* (*Graptocleptes bicolor*; Gil-Santana et al. 2013). This genus has been considered to be closely related to *Hiranetis* (Stål 1872, Champion 1898, Gil-Santana 2015). The male genitalia of *Hiranetis atra* showed similarities to those of *Graptocleptes bicolor*, such as: pygophore with a subtriangular rounded apex (medial process); parameres similar in shape and somewhat similar in vestiture; dorsal phallothecal plate suboval in shape, with apical margin almost transverse, straight; and endosoma wall mostly minutely spiny. On the other hand, the shape of the struts is quite different, and the pattern observed in *Hiranetis atra* (Figs 12–13) may possibly be revealed as characteristic of this species, since the struts pattern has been shown to be useful with regard to the taxonomy of other Neotropical Harpactorini (e.g. Hart 1972, 1986, 1987, Zhang 2012). Interestingly, however, asymmetry on the apical portion of the median arms of the struts was recorded in the present study (Figs 12–13). No similar previous record could be found. If more specimens were to be observed in the future, it would be possible to ascertain whether this was an isolated anomaly or a real feature of the species. Thus, at least for the moment, and as stated in all the studies previously cited, the features of the male genitalia of *Hiranetis atra* that should specially be taken into consideration for future comparative purposes are the subtriangular rounded medial process of the pygophore (Fig. 7), the suboval shape of the dorsal phallothecal plate, with an apical margin that is almost transverse (Fig. 12), and the shape and “design” of the struts (Figs 12–13).

#### Distribution.

Colombia (Stål 1872, Maldonado 1990).

#### New records.

Costa Rica, Ecuador.

### Hiranetis braconiformis

Taxon classificationAnimaliaHemipteraReduviidae

(Burmeister, 1835)

Myocoris braconiformis Burmeister, 1835: 226 [description]; Burmeister 1838: 107 [redescription]; Stål 1866: 295 [citation]; Walker 1873b: 129 [catalog]; Wygodzinsky 1949: 40 [catalog].
Hiranetis braconiformis ; Stål 1859: 371 [citation]; Stål 1872: 82 [redescription]; Lethierry and Severin 1896: 178 [catalog]; Champion 1898: 281 [included comments on color and morphological features], Tab. XVII [Figures 8, 8a, 9]; Maldonado 1990: 218 [catalog]; Maldonado and Lozada 1992: 165 [citation]; Froeschner 1999: 206 [catalog]; Gil-Santana et al. 2013: 348 [citation].
Myocoris pompilodes Burmeister, 1838: 106 [description]; Champion 1898: 281 [as a junior synonym of Hiranetis braconiformis].
Hiranetis pompilodes ; Stål 1859: 371 [citation]; Stål 1872: 82 [diagnosis]; Champion 1898: 281 [as a junior synonym of Hiranetis braconiformis]; Wygodzinsky 1949: 40 [catalog, as a valid species].
Myocoris pompiloides [*sic*]: Walker 1873b: 129 [catalog]; Maldonado 1990: 218 [catalog, as a junior synonym of Hiranetis braconiformis].
Hiranetis pompiloides [*sic*]: Lethierry and Severin 1896: 178 [catalog]; Maldonado 1990: 218 [catalog, as a junior synonym of Hiranetis braconiformis].

#### Material examined.

*Myocoris braconiformis*, female, “typus”, labels: 2777 / *Braconiformis*, N., Stoll. Cim. t. 21.f.147 [green label] / Pará, Sieber [green label] / Typus [red label]; *Myocoris pompilodes*, female, “typus”, labels: 2771 / *Pompilodes*, N. [green label] / Cameta, Sieber [green label] / Typus [red label]; *Myocoris pompilodes*, male, “allotypus”, labels: 2771 / * *Hiranetis pompilodes* Burm., ♂, Allotypus / Cameta, Sieber [green label] / Allo-Typus [red label] (ZMHB).

**Figures 18–24.**
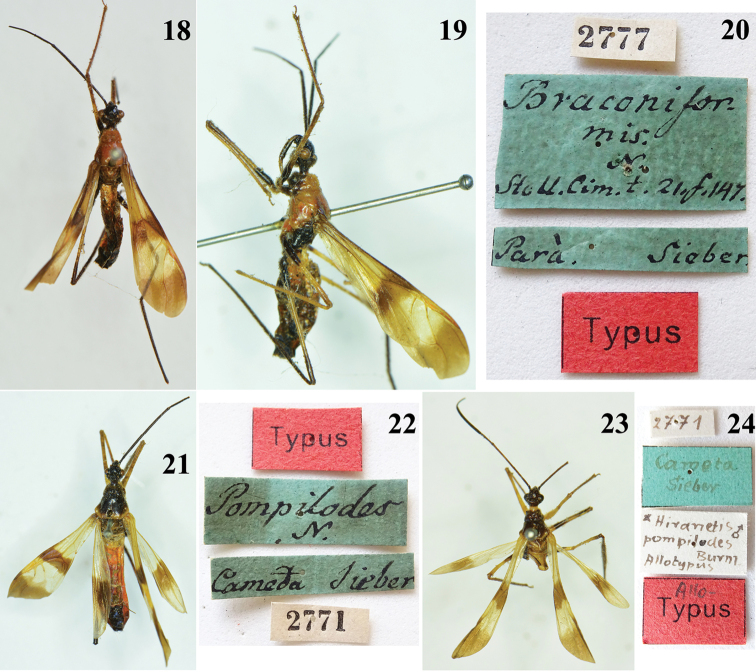
**18–20** *Myocoris braconiformis* Burmeister, female, “Typus”, deposited in ZMHB **18** dorsal view **19** lateral view **20** labels. **21–24** *Myocoris pompilodes* Burmeister, type specimens deposited in ZMHB **21**–**22** female, “Typus” **21** dorsal view **22** labels **23–24** male “allotypus” **23** dorsal view **24** labels.

The female “typus” of *Hiranetis braconiformis* (Burmeister), described from “Para” (Burmeister 1835, 1838), is deposited in ZMHB (Figs 18–20). This region (“Pará”) is today a state in the northern region of Brazil, within the Amazonian region of South America.

The female “typus” and a male “allotypus” of *Hiranetis pompilodes* (Burmeister), from “Cameta”, are also deposited in ZMHB (Figs 21–24). Because the original description (Burmeister 1838) did not designate a holotype, it is better to consider all the type specimens to be syntypes. In the male type, the distal portion of the abdomen is missing (Fig. 23). Although Burmeister (1838) had mentioned that “Cameta” was in “South Brazil”, the only locality with this name in Brazil is the municipality of “Cametá” in the same northern state of Pará, from which *Hiranetis braconiformis* was described. It is possible that all these specimens were collected in the same region (Pará) and even during the same period, since on all the labels, the name “Cameta” was followed by the name “Sieber” and at least those of the female syntype were apparently handwritten by the same person (Figs 20, 22). As a matter of fact, Friedrich Wilhelm Sieber was a servant and preparator of Johann Centurius Count von Hoffmannsegg, who obtained permission from the King of Portugal to send him to Brazil to collect insects. Leaving Lisbon in 1801, Sieber went to the province of Pará, where he remained 12 years, collecting in different parts of this province, including Cametá (Papavero 1971). Friedrich W. Sieber did not collect in other regions of Brazil and remained in Amazonia throughout this period (Papavero 1971), which reinforces the preceding assertion.

All of these points may be important in ascertaining the type locality of these taxa and are particularly relevant because *Hiranetis pompilodes* was subsequently considered by Champion (1898) to be a junior synonym of *Hiranetis braconiformis*.

With the exception of the mention of the length, the descriptions of *Hiranetis braconiformis* and *Hiranetis pompilodes* emphasized only their coloration (Burmeister 1835, 1838). Stål (1872) stated that the two taxa were very similar and *Hiranetis pompilodes* differed from *Hiranetis braconiformis* through the coloration of the thorax (blackish, except at its margin), coxae, trochanters and basal portion of fore femora (yellowish and not blackish).

Champion (1898) considered *Hiranetis pompilodes* to be a junior synonym of *Hiranetis braconiformis*. He reported that he had examined “a long series” of *Hiranetis braconiformis*, stating that it varied in “the colour of the pronotum and also to a certain extent in that of the femora. In many of the specimens the pronotum is entirely rufo-testaceous (*braconiformis*, Burm.); but in others (...) it is partly or entirely black, the basal margin or a subtriangular patch on the disc behind being pale in some examples (*pompilodes*, Burm.).” Champion (1898) also recorded variation in coloration of the femora, which are sometimes narrowly (fore femora) to broadly (mid and hind femora) black basally; “the hind pair have the apex broadly, and rarely a median ring, fuscous or black”, while the mid femora are often infuscate apically. In his figures of a pair of this species, he highlighted the variation in color amongst specimens from the same locality.

The fact that Champion (1898) recorded the color variation among specimens from the same locality, which had been attributed by Burmeister (1838) and Stål (1872) to *Hiranetis braconiformis* and *Hiranetis pompilodes*, may be considered to be arguments in favor of both the historical evidence that the types of these taxa must have been collected in the same region (Brazilian state of Pará) and the assumption that they belong to the same species as stated by Champion (1898).

On the other hand, Champion (1898) apparently did not examine any type specimens of these taxa, or any specimen from Brazil. He also did not mention how many specimens formed his “long series”, or whether there might be any other sexual differences besides the third antennal segment thickened at its base. Moreover, he did not take into account any features other than coloration when commenting on the synonymy between *Hiranetis braconiformis* and *Hiranetis pompilodes*.

Subsequently, Wygodzinsky (1949) still listed *Hiranetis pompilodes* as a valid species in his catalogue, while Maldonado (1990) considered it to be a junior synonym of *Hiranetis braconiformis*.

#### Distribution.

Brazil (state of Pará, Amazonian region) (Burmeister 1835, 1838), Mexico, Guatemala, Costa Rica, Panama and Guyana (Champion 1898).

### Hiranetis membranacea

Taxon classificationAnimaliaHemipteraReduviidae

Spinola, 1840

Hiranetis membranacea Spinola, 1840: 113–114 [description]; Lethierry and Severin 1896: 178 [catalog]; Wygodzinsky 1949: 40 [catalog]; Maldonado 1990: 218 [catalog]; Gil-Santana et al. 2013: 348 [citation].
Myocoris membranaceus ; Herrich-Schäffer 1848: 43 [redescription], Tab. CCLXI [Figure 811].
Myocoris barbipes Burmeister, 1838: 107 [description]; Stål 1866: 295 [citation]; Stål 1872: 82 [as a junior synonym of Hiranetis membranacea]; Walker 1873b: 129 [catalog, as a valid species]; Lethierry & Severin 1896: 178 [catalog, as a junior synonym of Hiranetis membranacea]; Maldonado 1990: 218 [catalog, as a junior synonym of Hiranetis membranacea].
Hiranetis barbipes ; Stål 1859: 371 [citation in text, with footnote: “= membranaceus Spin.; H. Sh.”]; Wygodzinsky 1949: 40 [catalog, as a junior synonym of Hiranetis membranacea].

#### Material examined.

*Myocoris barbipes*, female, “typus”, labels: 2772 / *barbipes*, two unrecognizable markings, &, ♀. / Bras. r. Olf. [green label] / Typus [red label] (ZMHB).

The description of *Hiranetis* mentioned some structural features, and also that their tibiae are all hairy (Spinola 1840). This was followed by the description of *Hiranetis membranacea*, based on one or more females and males from Brazil, without ascribing any specific locality to the specimens described. The antennae, body and legs of this species were recorded as black; the hemelytra as entirely membranous, blackish, often slightly darker at their base to their end, but all transparent: a large yellow spot, on three-quarters of their length on outer edge, and a smaller, hyaline, also on the external borders. Single measurements were attributed to the species (“m. long 9. lign. Larg. 2. Lign.”; approximately 20.3 and 4.5 mm, respectively).

After making this short description, Spinola (1840) commented that *Hiranetis membranacea* did not seem to be rare in South America and often showed variation: 1 - in the coloration of the thorax and abdomen, which were black, brown or even testaceous; 2 - in the legs, which could have yellowish annulus or be entirely yellowish; 3 - in the coloration of the hemelytra, which could be lighter-colored or hyaline, even in the basal portion, in some specimens; 4 - in the size, which could be half of or a third smaller.

However, he concluded by stating that the intermediary specimens that he had at hand left no doubt in his own mind regarding the unity of the species.

Unfortunately, it was not possible to locate any type specimen of *Hiranetis membranacea*. The material described by Massimiliano Spinola (1780–1857) is in his collection, which is deposited in the “Museo Regionale di Scienze Naturali”, Turin, Italy (Schuh and Slater 1995). More than a decade ago, when looking for a type of other species described by M. Spinola (see Forero and Gil-Santana 2003), I contacted its [former] curator, Dr Mauro Daccordi, who kindly donated the catalogue of Spinola’s hemipterological collection (Casale 1981), clarifying that all extant specimens were listed there. There is no reference to any specimens of *Hiranetis membranacea* in it. It is worth mentioning that after M. Spinola’s death (1857), his hemipterological collection remained in Tassarolo Castle until its acquisition by Museo Regionale di Scienze Naturali of Turin in 1979 (Casolari and Moreno 1980, Casale 1981). Taking into account “the precarious conditions the Collection was exposed for over a century” (Casale 1981), the types of *Hiranetis membranacea* can be considered lost. Nonetheless, at the end of 2015, I contacted the current curator of the Museum, Dr Marinella Garzena, who also kindly confirmed that no specimens of this species are present there. Therefore, it must be assumed that no type specimens of *Hiranetis membranacea* Spinola exist anymore.

**Figures 25–27.**
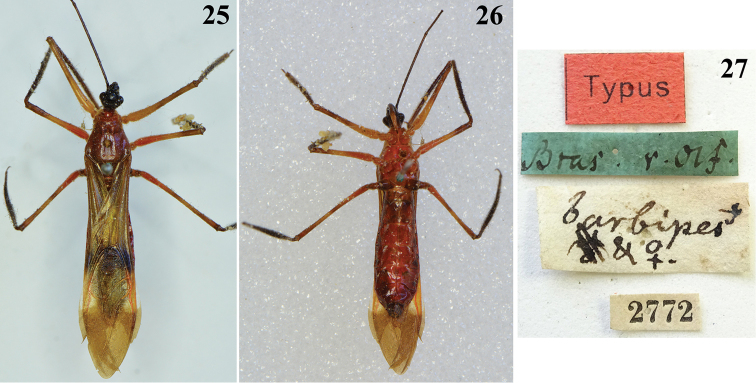
*Myocoris barbipes* Burmeister, female, Typus”, deposited in ZMHB. **25** dorsal view **26** ventral view **27** labels.

*Myocoris barbipes* was considered to be the largest species among several other species that were included in *Myocoris* Burmeister, 1835, at that time (Burmeister 1838). This species was recorded as coming from “Rio Janeiro” (Burmeister 1838). Its female “typus” is deposited in ZMHB (Figs 25–27). Regarding its type locality, “Rio Janeiro” (Burmeister 1838) may correspond to the current municipality of Rio de Janeiro or, because of the historical scenario at the beginning of the nineteenth century, more likely it should be extended to the state of Rio de Janeiro or even to some of the contiguous states in southeastern Brazil as they are currently delimited. In fact, the handwritten description on the green label attached to the type seems to read “Bras. r. Olf.” (Fig. 27). It is known that in 1816, Ignaz Franz Werner Maria von Olfers arrived in Rio de Janeiro with the Legation of Prussia to study Brazilian nature. He collected extensively in trips from the state of Rio de Janeiro to the contiguous states of Minas Gerais and São Paulo, and back to Rio de Janeiro, in the years 1818 to 1820. His collection, including insects, was then sent to museums in Vienna and Berlin (Papavero 1971).

Herrich-Schäffer (1848) provided a figure (habitus) and a short diagnosis of *Hiranetis membranacea* (as *Myocoris membranaceus*). The diagnosis referred only to color features: [general coloration] red; antenna, head, femora apices, tibiae and tarsi black; hemelytra pale yellowish with a median band and apex dark. He then commented on the variation in coloration and size, as had previously been recorded by Spinola (1840) for this species.

In a footnote, Stål (1859) mentioned *Hiranetis barbipes* (“= *membranaceus* Spin.; H. Sh.”). On the other hand, in Stål (1872), *Myocoris barbipes* was set as a junior synonym of *Hiranetis membranacea*. There was no mention of the reasons for attributing synonymy to these two species. However, with exception of Walker (1873), this was adopted in all the subsequent catalogues (Lethierry and Severin 1896, Wygodzinsky 1949, Maldonado 1990).

Stål (1872) recorded features of structure and vestiture in his diagnosis of the species of *Hiranetis*. However, it is unlikely that these will be helpful in ascertaining better characteristics of *Hiranetis membranacea*, so as to remove doubts regarding the validity of the synonymy that he proposed, and/or to provide better knowledge about the diagnostic features of all taxa discussed here. Firstly because he reported that he had examined a single specimen of *Hiranetis membranacea* from “Brasilia” [i.e., country of Brazil], which he stated was deposited in the Museum of Stockholm [“Mus. Holm.”]. Taking into consideration all the historical data on types of *Hiranetis membranacea*, there is no evidence that this specimen could be a type. In this case, it becomes clear that he did not examine the type of *Myocoris barbipes* that is still extant and is deposited in Berlin (ZMHB; Figs 25–27), even though he placed *Myocoris barbipes* as a junior synonym of *Hiranetis membranacea*. Similarly, regarding *Hiranetis braconiformis*, he also cited “Mus. Holm.”, thus denoting that he probably used other specimen(s) but not the type (also deposited in ZMHB; Figs 18–20) to define the features of the latter species. Secondly, among those features, some are common to other species of *Hiranetis* and coincide with the diagnosis of the genus, or may even be common to species of other genera. Some other features are known to vary among specimens and the possibility of inter-individual variation was probably not taken into consideration at that time. Thirdly, as discussed below, the recorded variations in *Hiranetis membranacea* (Spinola 1840) and *H.* [cf.] *braconiformis* (Champion 1848), the similarities in coloration between them and the absence of records of other or better features of each of them when they were originally described (Spinola 1840, Burmeister 1835, 1838) make any identification imprecise. This compromises the diagnosis of *Hiranetis membranacea* and *Hiranetis braconiformis* furnished by Stål (1872), because it seems that he did not examine any type specimens of these species.

#### Distribution.

Brazil (Spinola 1840, Burmeister 1838, Herrich-Schäffer 1848, Maldonado 1990).

## Discussion

*Hiranetis atra* can be separated from the other species of the genus by its coloration, which is predominantly blackish, including the hemelytra, which are entirely dark (Figs 1–3, 5–6).

Otherwise, while all other currently valid species have the pattern of yellowish or straw-colored hemelytra, with a median, transverse band and a dark apex (Figs 18–19, 21, 23, 25), the limits or validity of these species are uncertain. It is possible that they could be variations of a single species or could be two or more species.

Taking in account the variation in *Hiranetis membranacea*, in relation to its description by Spinola (1840), as commented on above, it is possible that among the specimens of the type series, more than a single species could have been present. Unfortunately, this hypothesis is no longer verifiable, because these specimens have been lost.

As discussed above, the previous statements regarding synonymies between taxa of *Hiranetis* (Stål 1872, Champion 1898) needs to be better reviewed, because none of them were based on examination of type specimens and they took in account only coloration (Champion 1898) or a few structural features that were not mentioned in the original descriptions, with feeble or no taxonomic value, verified in only a few specimens (Stål 1872). Moreover, the reliability of the identification of the specimens studied by these authors (Stål 1872, Champion 1898) may be considered doubtful.

A better record of size, including possible sexual variation, and studies on structural features, particularly the male genitalia, and possibly a molecular approach, could help or be determinant in defining the taxonomy of *Hiranetis braconiformis*, *Hiranetis barbipes*, *Hiranetis membranacea*, and *Hiranetis pompilodes*.

However, it seems that such studies on the type specimens will be impossible. In addition to the loss of types of *Hiranetis membranacea*, two of the extant types are females and in the only male, the distal portion of the abdomen is missing, and consequently the genitalia is no longer available for examination.

Therefore, in order to resolve the taxonomy of *Hiranetis* spp. a taxonomic review of the group should be done in the future, including the study of a new series of specimens.

## Supplementary Material

XML Treatment for Hiranetis

XML Treatment for Hiranetis atra

XML Treatment for Hiranetis braconiformis

XML Treatment for Hiranetis membranacea
